# Late-Onset Mitral Valve Prosthesis Dehiscence With Severe Paravalvular Leak—Infectious Versus Noninfectious Etiology Dilemma: A Case Report

**DOI:** 10.1016/j.cjco.2024.07.009

**Published:** 2024-07-22

**Authors:** Nouhaila Lahmouch, Raid Faraj, Oualid Kerrouani, Asmae Bouamoud, Jamila Zarzur, Mohamed Cherti

**Affiliations:** Department of Cardiology B, Ibn Sina University Hospital, Mohammed V University, Rabat, Morocco

**Prosthetic paravalvular leakage stems from imperfect sealing between the prosthetic valve and the surrounding annulus, leading to backward blood flow. While often detected early after surgery, occurrences can emerge years later. We describe a 47-year-old woman, 12 years after aortic and mitral valve replacement, with hemolytic anemia. Echocardiography revealed mitral valve prosthesis dehiscence and severe paravalvular leakage. Although imaging suggested late-stage infectious endocarditis, intraoperative examination ruled it out. Reinforcing prosthesis sutures yielded positive results, underscoring diagnostic challenges and the need for diverse imaging modalities in managing late paravalvular leaks.**

Mitral paravalvular leak (PVL) is a recognised but rare complication after mitral valve replacement, occurring in about 7% to 17% of cases and being twice as common as with aortic prostheses.^1^ It can result from tissue calcification, improper valve sizing, infection, and tissue fragility. Moderate to severe cases more than double the risk of all-cause mortality.^1^ Consequently, managing mitral PVL necessitates a careful approach, weighing the potential risks of intervention against the benefits of symptom alleviation and complication prevention. What makes the present case unique is the occurrence of a significantly delayed onset of mitral PVL, alongside a discrepancy in etiology between imaging findings and perioperative assessment. This case report aims to clarify mitral PVL complications, highlighting clinical challenges and the need for a multidisciplinary approach for optimal outcomes.

## Case Presentation

A 47-year-old patient with no cardiovascular risk factors, but a history of rheumatic mitro-aortic disease and childhood acute rheumatic fever, underwent double mechanical prosthesis replacement in 2011. When she came to us recently, she had experienced worsening exertional dyspnoea (New York Heart Association stage II) and fatigue for more than a year. On admission, she had a heart rate of 98 beats/min and blood pressure 120/70 mm Hg, with no signs of heart failure. The electrocardiogram and chest X-ray were normal. Biologically, she presented with a normochromic normocytic regenerative anemia hemoglobin level of 8.9 g/dL (reference range 12.1-15.1 g/dL), with a reticulocyte count of 278,000/mm^3^, white blood cell count 4900/mm^3^ (reference range 4500-11,000/mm^3^), lactate dehydrogenase level 2000 U/L (reference range 140-280 U/L), haptoglobin level below the detection threshold, and normal total bilirubin level with a negative Coombs test. The blood smear showed a very high number of schistocytes, suggesting hemolytic anemia.

**Figure 1:**
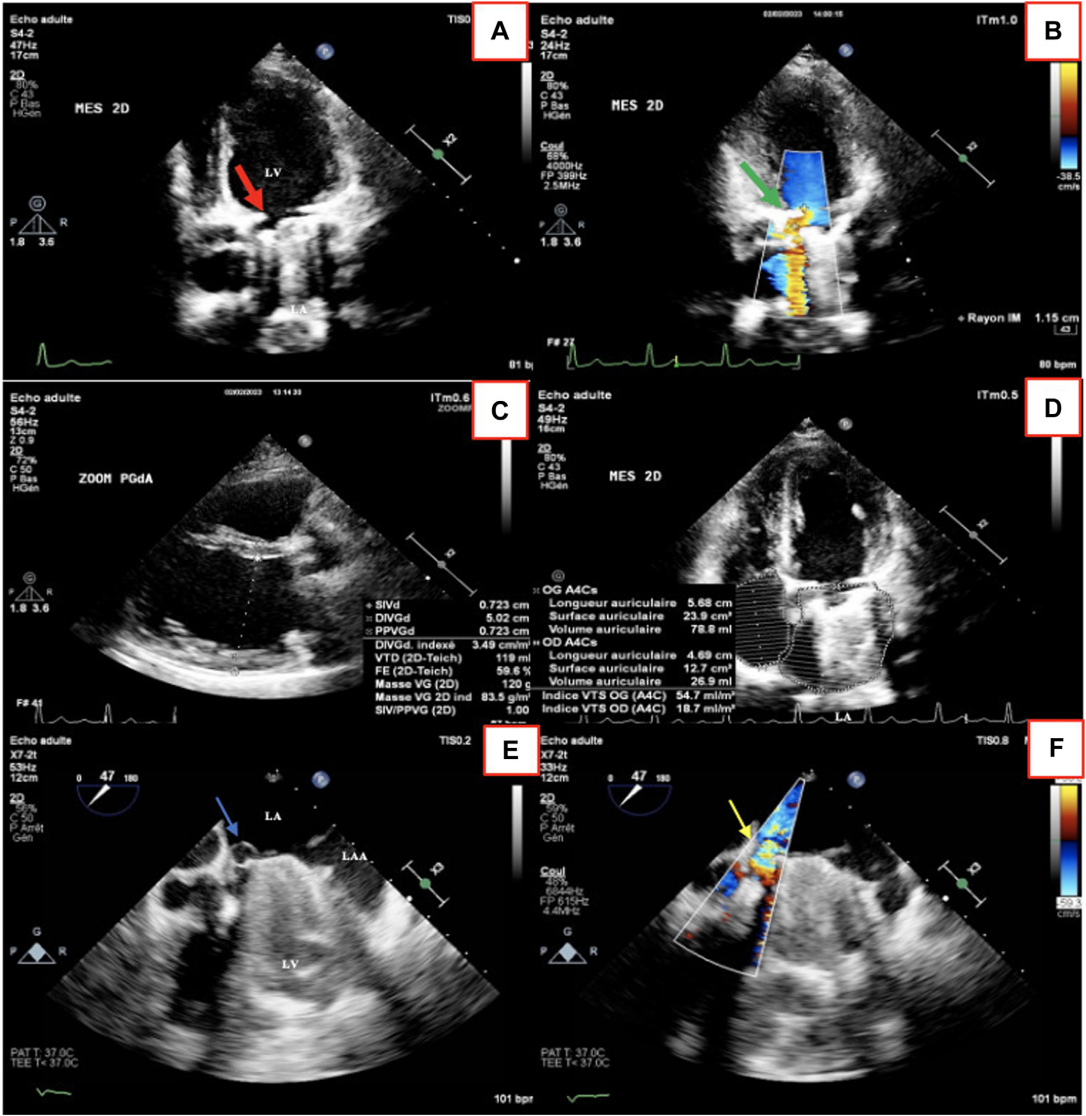
Multimodal echocardiographic assessment. (**A**) Transthoracic echocardiography (TTE) modified 4-chamber view, showing a mechanical bileaflet prosthesis in the mitral position with partial prosthesis detachment in the septal region (**red arrow**). (**B**) TTE modified 4-chamber view with color Doppler analysis, showing a severe paravalvular regurgitation jet (**green arrow**). (**C**) TTE parasternal long-axis view, showing a dilated left ventricle secondary to severe mitral regurgitation. (**D**) TTE 4-chamber view, showing a dilated left atrium secondary to severe mitral regurgitation. (**E**) Transesophageal echocardiography (TEE) modified 4-chamber mid-esophageal view, revealing a partial dehiscence of the mitral prosthesis, with mobile elements bent around the exterior of the annulus and protruding into the left atrium, suggesting the presence of vegetations or suture threads (**blue arrow**). (**F**) TEE with color Doppler analysis, showing a severe paravalvular regurgitation jet (**yellow arrow**). LA, left atrium; LAA, left atrial appendage; LV, left ventricle.

Transthoracic echocardiogram (TTE) showed a dilated left ventricle with preserved systolic function, an ejection fraction of 57%, a mechanical bileaflet prosthesis in the aortic position functioning well, nonleaking and nonstenotic, and a mechanical bileaflet prosthesis in the mitral position, nonstenotic but exhibiting significant PVL with partial prosthesis detachment (PISA radius of 11 mm, regurgitant orifice area of 63 mm^2^, and regurgitant volume of 90 mL) (Fig. 1; Videos 1 and 2 , view videos online).

**Figure 2:**
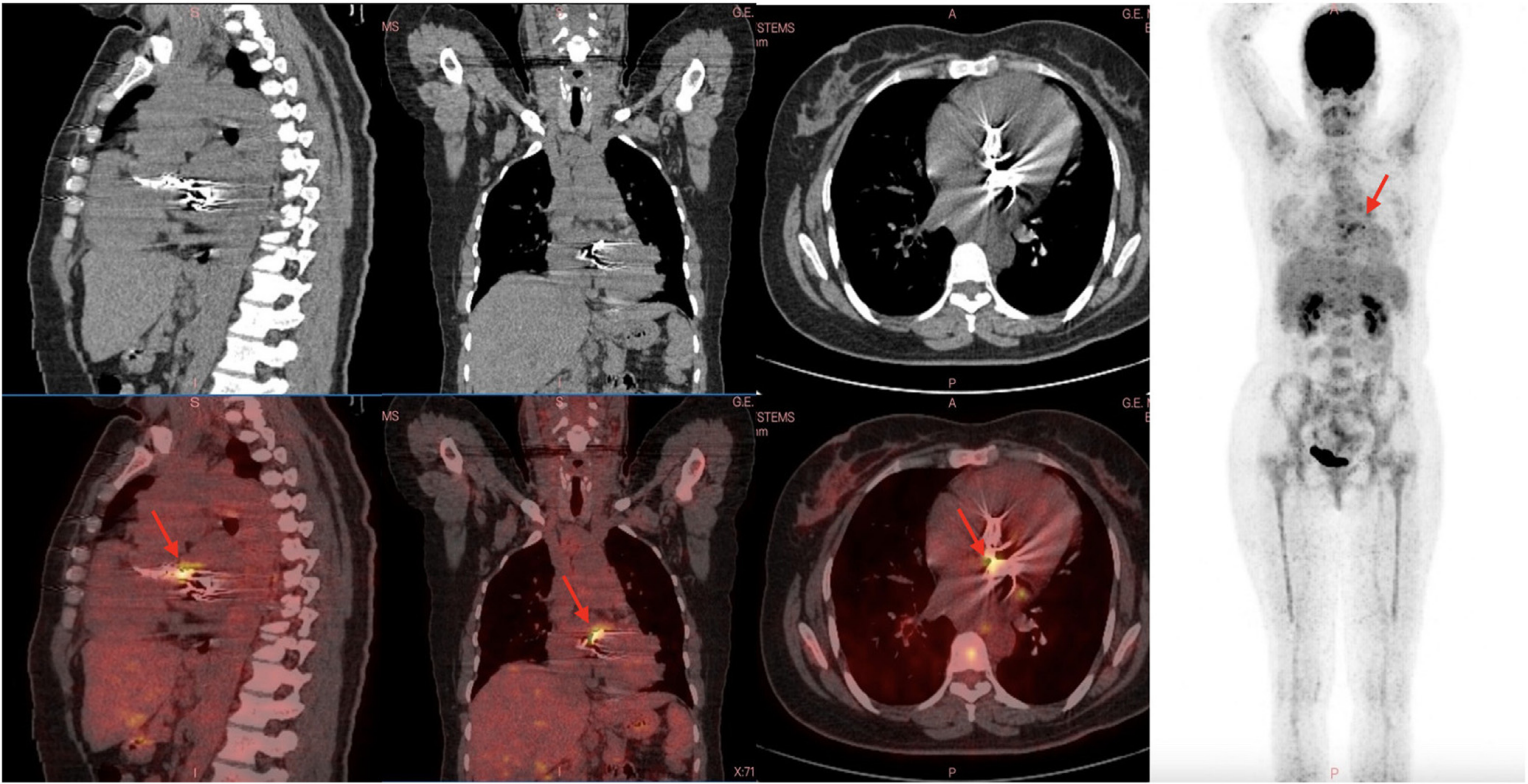
^18^F-Fluorodeoxyglucose positron emission tomography/computed tomography findings, indicating an abnormal increase in metabolic activity (uptake) specifically in the area surrounding the mitral prosthesis (**red arrows**). This heightened uptake suggests potential abnormal cellular activity or inflammation associated with the mitral prosthesis.

Suspecting infective endocarditis, we performed transesophageal echocardiography (TEE), which revealed mitral prosthesis dehiscence with a significant paravalvular leak, a 5 mm convergence zone, and multiple mobile elements, the largest being 8 × 2 mm, possibly indicating vegetations or suture threads protruding into the left atrium. (Fig. 1; Videos 3 and 4 , view videos online). Blood cultures were negative, with no systemic signs of sepsis. C-Reactive protein was 20 mg/L (reference < 5 mg/L), and procalcitonin remained negative after 1 week. Serologic tests for common causes of culture-negative endocarditis also were negative. Despite the lack of clinical or biological signs, ^18^F-fluorodeoxyglucose (FDG) positron emission tomography (PET)/computed tomography (CT) was performed owing to the suspicion of infective endocarditis, revealing abnormal uptake in the mitral prosthesis but not around the aortic prosthesis (Fig. 2). We concluded possible late infective endocarditis (1 major criter) on mechanical mitral prosthesis complicated with severe PVL and hemolytic anemia. Empirical antibiotic treatment with vancomycin and gentamicin was started and continued for 6 weeks.

Because of the presence of an extensive leak not susceptible to percutaneous closure, the decision was made to perform surgical treatment. During surgery, the mechanical valve was found to be partially dehiscent with multiple suture threads but no vegetations or abscesses. The decision was made to keep the existing mitral prosthesis, strengthen the sutures, and adjust its position on the mitral ring. The postoperative course was uneventful. The patient remained afebrile with a normal white blood cell count, negative C-reactive protein, and negative blood cultures. Postoperative TTE showed no vegetations and normal function of the repositioned valve. After 6 months of follow-up, the patient remained free from prosthetic valve-related events and symptoms of infection. TEE was performed postoperatively, confirming the proper function of 2 mechanical mitro-aortic prostheses with no leakage or stenosis. The previous PVL and mobile suture–related elements were resolved (Supplemental Fig. S1; Video 5 ).

## Discussion

Valvular heart disease (VHD) is increasingly prevalent owing to higher survival rates and an aging population, leading to more valve replacements and a rise in PVLs. Mitral PVLs are 3 times more common than aortic PVLs, especially after reoperations.^2^ PVLs have significant prognostic implications, with mitral PVLs linked to lower event-free survival rates compared with aortic PVLs. They result from incomplete sewing ring apposition, mainly due to suture dehiscence and factors such as tissue friability, limited suture space, annular calcium, infection, noncircular ring prosthetics, and technical challenges. Moreover, mechanical implants and continuous sutures, particularly in mitral prostheses, increase PVL risk.^2^ Some authors suggest that PVLs primarily occur within the first year after surgery.^2^ However, a 20-year follow-up study by Ho Young Hwang et al. in Korea,^3^ involving 1226 mitral valve replacement patients, found that only 1.9% experienced early major mitral PVL. In contrast, 6% developed major late PVL without evident infection, with some cases emerging up to 250 months after surgery. This delayed occurrence was particularly notable in elderly men and those with repeated operations, highlighting the need for extended postoperative monitoring.

The present case is unique, with severe PVL appearing 12 years after surgery in a young woman with no repeated operations. Diagnosing and characterising PVLs is challenging, often requiring various imaging techniques. TTE is the primary tool for assessing prosthetic valve structure and function, although its accuracy can be hindered by factors such as acoustic shadowing. Conversely, TEE, especially with 3-dimensional imaging, is considered the criterion standard, excelling in evaluating severity, valve annulus size, PVL localisation, and etiology. In our case, TTE and TEE together provided a thorough assessment of the PVL’s presentation and extent but did not definitively identify the cause.

Distinguishing between vegetations and suture threads can be challenging. Marco Gennari et al.^4^ reported a case of a 58-year-old woman with a late PVL attributed to monofilament suture rupture and confirmed during surgery. In contrast, our case involved suspected infectious endocarditis on a mechanical prosthesis. We utilized an ^18^F-FDG PET/CT scan, which revealed increased ^18^F-FDG in the mitral prosthesis, guiding the diagnosis toward an infectious origin. Several studies support this choice, showing the sensitivity of ^18^F-FDG PET/CT for prosthetic valve endocarditis (PVE) to range from 73% to 100% and the specificity from 71% to 100%, with positive predictive values of 67% to 100% and negative predictive values of 50% to 100%.^5^ Combining ^18^F-FDG PET/CT with the modified Duke criteria increases sensitivity from 52%-70% to 91%-97%, while maintaining specificity. Accordingly, the 2023 European Society of Cardiology guidelines for infective endocarditis include a class I B recommendation for using ^18^F-FDG PET/CT to detect valvular lesions and confirm PVE diagnosis. However, ^18^F-FDG PET/CT can produce false positives. A 2016 study by Ulises Granados et al.^6^ found 2 false-positive PVE cases where ^18^F-FDG PET/CT was performed 1 and 8 months after surgery. Similarly, Asbjørn Mathias Scholtens et al.^7^ highlighted scenarios leading to misdiagnosis. They noted mild to moderate ^18^F-FDG uptake near the prosthetic valve ring and/or the struts in 2 patients with aortic mechanical prostheses, later confirmed as non-PVE. This uptake was deemed to be a normal variant, possibly due to mild foreign body reactions or strain on the aortic wall.

Medical management is limited in patients with symptomatic PVLs, because it cannot address the root cause. Both the American College of Cardiology/American Heart Association 2020 and European Society of Cardiology 2021 guidelines recommend surgical repair as the preferred treatment for symptomatic PVLs. Transcatheter closure has shown favourable outcomes with minimal complications, but is not recommended for cases involving prosthesis instability, active endocarditis, or extensive PVLs affecting more than 30% of the valve circumference. In the present case, intraoperative findings did not indicate infectious endocarditis, underscoring the critical role of multidisciplinary collaboration within the heart team. Our approach—opting to reinforce sutures and reposition the prosthetic valve rather than replace it—proved successful. The patient demonstrated favourable progress over a 1-year follow-up period. However, the lack of a surgical specimen for histopathologic analysis to confirm the absence of endocarditis is a significant limitation of our case report.

**Novel Teaching Points**

• Noninfectious PVL can occur at later time points after surgery.
• Extended postoperative monitoring is important, because late-onset PVLs can manifest years after surgery.
• Multidisciplinary collaboration within the heart team is crucial for managing late PVLs effectively.

## Conclusion

This case highlights the diagnostic and treatment challenges of mitral PVLs. Accurate etiologic diagnosis requires multidisciplinary collaboration and advanced imaging techniques. Clinicians should remain vigilant for PVLs, even in patients presenting with new symptoms decades after surgery.

## Supplementary Material

Supplementary Figure S1

Supplementary Video 1 Transthoracic echocardiography apical 4-chamber view, revealing a mechanical bileaflet prosthesis in the mitral position with a partial prosthesis detachment in the septal region.

Supplementary Video 2 Color Doppler analysis in a transthoracic echocardiography modified apical 4-chamber view, revealing a severe paravalvular regurgitation jet.

Supplementary Video 3 Transesophageal echocardiography modified 4-chamber mid-esophageal view, revealing a partial dehiscence of the mitral prosthesis, with mobile elements bent around the exterior of the annulus and protruding into the left atrium, suggesting the presence of vegetations or suture threads.

Supplementary Video 4 Color Doppler analysis in a transesophageal echocardiography modified 4-chamber mid-esophageal view, showing a severe paravalvular regurgitation jet.

Supplementary Video 5 Postoperative transesophageal echocardiography findings. In the modified 4-chamber mid-esophageal view, a well positioned mitral prosthesis is observed, with no evidence of paravalvular regurgitation, and there is disappearance of the mobile elements.
