# Supplementary material for: Late-Onset Mitral Valve Prosthesis Dehiscence With Severe Paravalvular Leak—Infectious Versus Noninfectious Etiology Dilemma: A Case Report
Source: CJC Open. 2024 Jul 22;6(11):1255–8. doi: 10.1016/j.cjco.2024.07.009 (PMC11583854; doi:10.1016/j.cjco.2024.07.009)

<u>Supplemental Figure S1:</u> Post-operative TOE findings: In the modified four-chamber mid-esophageal view, a well-positioned mitral prosthesis is observed, with no evidence of paravalvular regurgitation, and there is disappearance of the mobile elements.

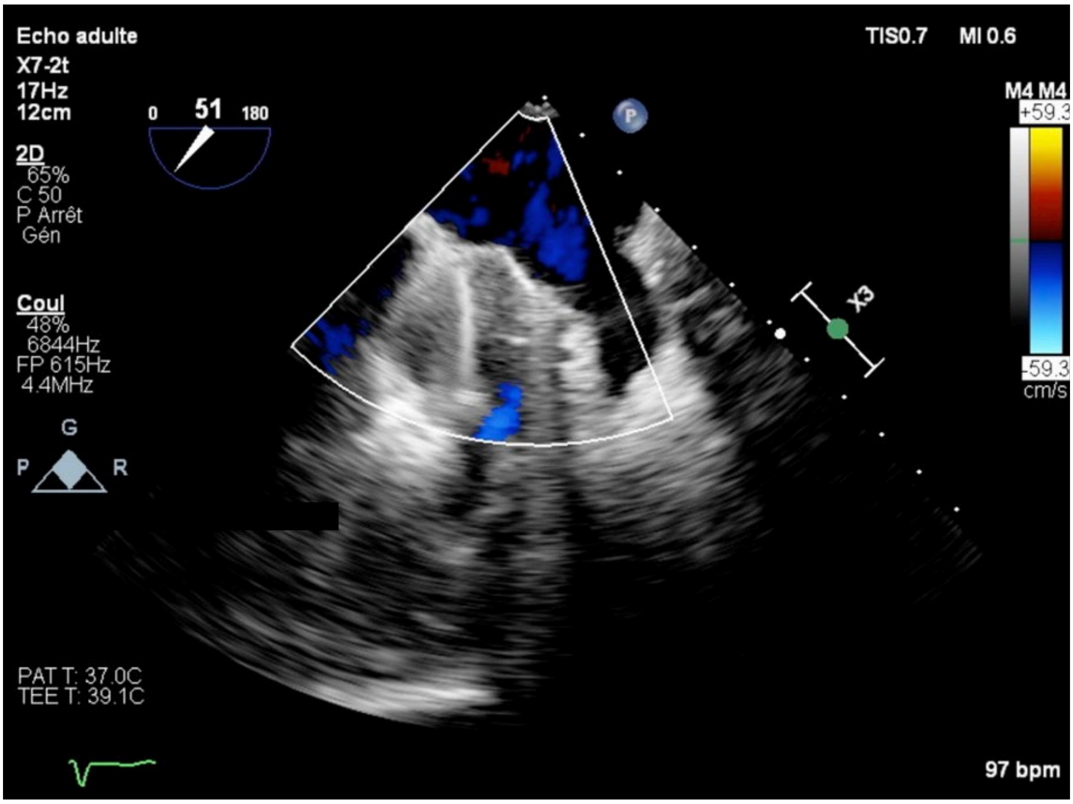

Supplement: Supplementary Figure S1 [file mmc1.pdf]
